# Decision‐Making Capacity and Awareness in People with Young‐Onset Alzheimer´s Disease

**DOI:** 10.1002/alz.089444

**Published:** 2025-01-03

**Authors:** Marcia C. N. Dourado, Nataie Souza, Tatiana Belfort, Marcela Moreira Lima Nogueira, Maria Alice Baptista, Maria Alice Baptista

**Affiliations:** ^1^ Federal University of Rio de Janeiro, Rio de Janeiro Brazil; ^2^ Federal University of Rio de Janeiro, Rio de Janeiro, Rio de Janeiro Brazil

## Abstract

**Background:**

There is a lack of research on differences between decision‐making capacity and awareness according to age at onset of dementia. We investigated the relationship between decision‐making capacity and awareness domains in people with young‐ (YOAD) and late‐onset Alzheimer´s Disease (LOAD).

**Method:**

A cross‐sectional study included 169 consecutively selected people with AD and their caregivers (124 people with LOAD and 45 people with YOAD).

**Result:**

People with YOAD were more cognitively impaired, but more aware of their cognitive deficits and health condition, with moderate effect sizes. All people with AD presented deficits in the domains of decision‐making capacity, with more impairment in understanding. There was a relationship between understanding and awareness domains, such that awareness was particularly important for decision‐making capacity in the YOAD group.

**Conclusion:**

Better awareness involved better understanding in the YOAD group. Clinically, our findings shed light on the need to consider the differences in the domains of awareness and their relationship with other clinical aspects such as decision‐making capacity according to age at onset of AD.

